# Contribution of C1 Biotechnology to the Achievement of the United Nations’ Sustainable Development Goals

**DOI:** 10.3390/bioengineering13050505

**Published:** 2026-04-27

**Authors:** Maximilian Lackner, Arabi Sivanesapillai, Dirk Holtmann

**Affiliations:** 1 CIRCE Biotechnologie GmbH, Kerpengasse 125, 1210 Vienna, Austria; m.lackner@circe.at; 2 Polymer Institute, Slovak Academy of Sciences, Dúbravská cesta 9, 845 41 Bratislava, Slovakia; 3 Institute of Process Engineering in Life Sciences, Karlsruhe Institute of Technology, Fritz-Haber-Weg 4, 76131 Karlsruhe, Germany; arabi.sivanesapillai@kit.edu

**Keywords:** C1 biotechnology, United Nations Sustainable Development Goals, gas fermentation, synthesis gas, bioeconomy, biofuels, bioplastics, alternative proteins, sustainability, bio-based building blocks

## Abstract

C1 biotechnology—bioprocesses that valorize one-carbon feedstocks such as CO_2_, CO-rich gases (blast furnace gas or synthesis gas), CH_4_ and CH_3_OH—has evolved from laboratory curiosity to industrial reality. In the quest to de-fossilize the chemical industry, the circular bioeconomy is widely seen as a solution. However, today it is still mostly based on primary agricultural feedstocks. Compared to thermochemical and catalytic processes, bioprocesses (fermentations) are carried out at ambient conditions, achieve high selectivities and good productivities. By decoupling fermentation from sugar-based substrates, gas fermentation of C1 substrates offers a scalable technology platform for producing biofuels, bioplastics, bio-based building blocks and alternative proteins, to name a few large-volume products. C1 platforms enable a circular, resource-efficient and virtually feedstock-independent bioeconomy that directly supports multiple United Nations Sustainable Development Goals (SDGs). In this article, we analyze the current technological landscape and discuss the (potential) impact of C1 routes on key SDGs using recent research advances and commercial case studies.

## 1. Introduction

In 2015, the United Nations adopted the 2030 Agenda for Sustainable Development (UN, 2025), introducing 17 Sustainable Development Goals (SDGs) as a universal call to action to end poverty, protect the planet, and ensure prosperity for all [1]. These goals address a broad spectrum of pressing social, economic, and environmental challenges, from climate action (SDG 13) and affordable clean energy (SDG 7) to sustainable consumption and production (SDG 12) and zero hunger (SDG 2). Yet, a decade into their implementation, progress remains uneven and insufficient. Industrial emissions continue to rise, biodiversity loss is accelerating, and uneven progress among regions is observed. In this context, transformative technologies are urgently needed to decouple economic growth from environmental degradation and to drive sustainable innovation across sectors.

Biotechnology has long been identified as a cornerstone of the emerging bioeconomy, offering renewable and circular alternatives to fossil-based systems. However, most established biotechnological processes rely on sugar-based feedstocks such as glucose, sucrose, or starch—substrates which are derived from agricultural crops and thus compete with food production, require large amounts of arable land and freshwater resources, and generate significant greenhouse gas (GHG) emissions when grown intensively [2]. This “sugar economy” creates trade-offs that constrain the scalability and true sustainability of many first-generation (1G) bioprocesses. We have seen the inability of 1G biofuels to be produced sustainably, due to their large implemented volumes, which trigger unexpected and undesired effects at a large scale, including land use changes. Other replacements and substitutes for fossil-derived materials such as bioplastics have not yet been scaled up sufficiently to cause true feed and food competition. However, the scaling potential of first-generation bioproduct processes is limited as soon as they start yielding volumes equivalent to a few percent of today’s bulk fossil-derived products. For example, as a rough calculation: There are ~400 Mio. tons/year of plastic production. At ~10 tons of starch per ha and year productivity and ~2.5 tons of required starch per ton of bioplastics, one would need 1 Mio. km^2^ of arable land to produce that volume of material. That is roughly the total land area dedicated to soy production globally, which shows that it is impossible to simply switch from fossil resources to sugar/starch from primary agricultural production.

C1 biotechnology offers a fundamentally different paradigm. Instead of relying on multi-carbon sugars, it exploits single-carbon (C1) substrates—such as carbon dioxide (CO_2_), carbon monoxide (CO), methane (CH_4_), formic acid (HCOOH), or methanol (CH_3_OH)—as the sole carbon and energy source for microbial growth and product formation. Apart from methanol and formate, feedstocks in C1 biotechnology are gaseous; hence it is often referred to as “gas fermentation”. This field has seen rapid development over the past decade, driven by advances in synthetic biology, bioprocess engineering, and carbon capture technologies. CO_2_, from point sources and direct air capture, has been targeted as a substrate for chemical synthesis, where bioprocesses are an interesting route, using H_2_ as an energy source.

These C1 substrates are often derived from industrial waste gases, renewable electricity (via power-to-X technologies, e.g., CO_2_ to CH_4_ methanation), or synthetic natural gas from agricultural waste, landfills or (deserted) coal mines, enabling the capture and valorization of emissions that would otherwise contribute to global warming. Microorganisms capable of C1 metabolism, including acetogens, methanotrophs, formatotrophs and methylotrophs, are now being selected, adapted and engineered to produce a wide range of value-added compounds, including fuels, bioplastics, (bio-based) building blocks as base chemicals and protein-rich biomass. Beyond direct gas-to-product conversions, C1 biotechnology also enables two-stage, coupled processes, e.g., in acetate-mediated single-cell protein (SCP) production, acetogens convert CO_2_ and H_2_ to acetate, which is subsequently consumed by aerobic microorganisms (e.g., *Yarrowia lipolytica*) to generate protein-rich biomass [3,4,5]. Such coupled processes improve carbon fixation and can reduce land and water requirements by up to 10–100-fold compared to conventional animal or plant-derived proteins [6,7]. While several modeling studies indicate that these systems can reach very low or even negative life-cycle emissions when powered by low-carbon electricity (e.g., −1.1–13 kg CO_2_-eq/kg_protein_ for formate-, acetate-, and ethanol-based systems), their current energy demand (25–56 kWh/kg_Protein_) still exceeds that of conventional protein sources such as soybean or raw beef meat. This highlights that future improvements in energy efficiency and access to renewable electricity will be crucial for realizing the full sustainability potential of coupled C1-based protein production.

In spite of the advantages that the C1 platform offers and the advances that have been achieved, several technical challenges remain, and these affect scalability and process economics: maintaining high gas–liquid mass transfer without excessive energy input, handling of flammable gases in pressurized reactors, preventing cross-contamination between units in coupled processes, as well as managing product inhibition at high titers. Addressing these concerns will require new reactor designs, continued progress in strain engineering and innovation in process design to optimize titer, rate and yield to ensure profitability.

**Figure 1:**
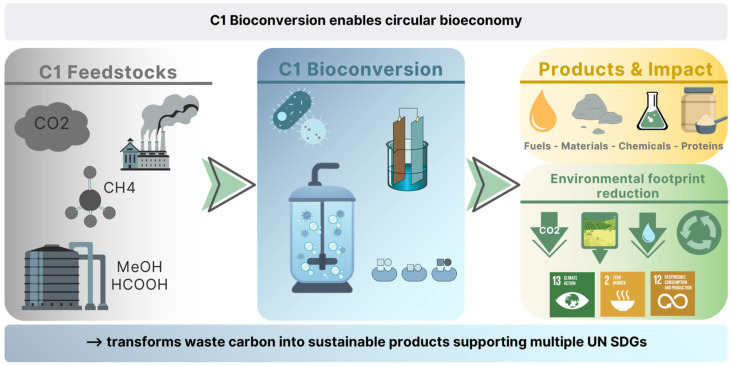
This text outlines the basic concepts of how C1 biotechnology could contribute to achieving the United Nations’ Sustainable Development Goals.

Based on a 2023 article [8] on the contribution of enzyme catalysis to the SDGs, this paper explores the contribution of C1 biotechnology to the realization of the SDGs, assessing the state-of-the-art, industrial applications, environmental performance, and strategic potential of these processes. In our article, we focus on gas fermentation and methanol-based bioprocesses as mature examples and discuss their alignment with specific SDGs. In particular, we highlight how C1 platforms can improve resource efficiency, reduce GHG emissions, and generate target products of interest such as alternative protein sources. By mapping technological advances to global sustainability targets, we aim to position C1 biotechnology as a key enabler of a non-sugar circular bioeconomy that supports equitable and environmentally sound development. The main aspects of the review are summarized in Figure 1.

In the ten years since the adoption of the United Nations’ 2030 Agenda for Sustainable Development [1], progress toward achieving the 17 SDGs has been mixed, and in many domains, alarmingly insufficient. While some targets—such as access to digital communication or reduction in extreme poverty—have seen measurable improvement, many core environmental and systemic goals are significantly off track. According to the 2024 UN report “The Sustainable Development Goals Report 2024”, over half of the SDG indicators are either stagnating or regressing, particularly those related to climate action (SDG 13), life on land (SDG 15), responsible consumption and production (SDG 12), and zero hunger (SDG 2). The individual SDGs are seen as equally important, and advancement is needed in each of them. The reasons for the lack of progress are multifactorial. Global population growth, geopolitical instability, the persistent dependency on fossil resources and the ever-growing absolute consumption. Despite growing awareness and regulatory frameworks, global CO_2_ emissions hit new records almost every year [9], and the decoupling of economic growth from resource use remains elusive at scale [10,11]. Industrial production, agriculture, and transport still rely heavily on linear models of extraction, use, and disposal, creating significant barriers to achieving the SDGs within the 2030 timeframe, which is no longer far in the future. Moreover, the COVID-19 pandemic, climate-related disasters, and recent geopolitical tensions have further exposed the fragility of the progress achieved so far. These setbacks have diverted political and financial attention from long-term sustainability objectives, threatening to delay critical transitions in energy, food, water and materials systems, which are all highly interrelated [12].

It is therefore evident that incremental improvements are no longer sufficient. Reaching the SDGs within the 2030 timeframe requires systemic change and transformative technologies that can shift entire value chains toward circularity, resilience, and low-carbon operation. Within this context, C1 biotechnology emerges as a promising and powerful yet underutilized solution that directly addresses key SDG challenges—particularly those at the nexus of climate, resource use, industrial innovation, and food security. While renewable energy deployment and improved energy efficiency remain central pillars of the transition, C1 biotechnology differs from these approaches by enabling direct transformation of carbon emissions into value-added products.

As we have entered the final five-year window before 2030, the question “quo vadis?”—where are we going?—demands a critical examination of both policy and technology. It also calls for the alignment of industrial innovation with global sustainability priorities. In the following sections, we explore how C1 biotechnology can serve as a strategic contributor to regaining momentum toward the SDGs and closing the implementation gap that has widened over the past decade. Early technological success was achieved, e.g., by the Pruteen™ plant in the UK, where methanol was converted into SCP at an industrial scale [13]. While this project failed economically due to the availability of cheaper soy-based proteins, in recent years, the first companies have successfully built large-scale gas fermentation installations, e.g., LanzaTech, converting CO into ethanol [14].

## 2. Microbial Platforms and Metabolic Strategies

A broad range of native metabolic pathways enable both aerobic and anaerobic microorganisms to grow on C1 substrates. In recent years, this list has been further expanded through the introduction of synthetic C1-fixation routes into conventional heterotrophic organisms such as *Escherichia coli* to overcome limitations associated with native C1 pathway organisms [15]. Microbial platforms for C1 conversion and associated metabolic engineering strategies have been reviewed extensively and provide detailed insights into native and synthetic C1 assimilation pathways, as well as strain engineering approaches [16,17,18,19,20,21]. The following groups of microorganisms dominate the current C1 biotechnology landscape:

### 2.1. Anaerobic C1 Assimilation

Acetogenic bacteria (e.g., *Clostridium ljungdahlii*, *Clostridium autoethanogenum*) utilize the Wood–Ljungdahl pathway, nature’s most energy efficient C1 fixation pathway, to fix CO_2_ and CO into acetyl-CoA, a central precursor for various metabolic products [22,23]. Acetogens have gained increasing attention in the field of C1-biomanufacturing due to their high carbon and electron efficiency, although their metabolism operates close to the thermodynamic limit with low ATP yields [24,25]. These organisms are mostly used in gas fermentation processes fed with syngas or industrial waste gases. Syngas today is mostly obtained from coal, but can also be attained from biogenic sources such as various waste biomass streams, including agricultural residues (e.g., rice straw, corn stover), forestry residues, municipal solid waste, and industrial by-products such as black liquor [26]. The CO/H_2_ ratio can be tuned by the choice of the gasification agent [27]. Besides syngas, certain species such as *Acetobacterium woodii* are also capable of efficiently utilizing liquid C1 substates such as formate and methanol [28]. To date, commercialization has largely focused on ethanol production [29], but recent developments in strain engineering and system-level characterization are opening the possibility to produce a variety of products. Some of the most relevant products are acetate, ethanol, 2,3-butanediol and butyrate, but genetic modifications can also be used to produce others, such as acetone and butanol [30].

Methanogenic archaea (methanogens) have the unique ability to produce methane as the primary product of their metabolism for energy production and growth [20]. They are limited to only a few substrates, all of which are converted to methane as the main product, which is a promising candidate to replace fossil fuels. Compounds that serve as substrates for methanogenic bacteria are, e.g., H_2_/CO_2_, formate, acetate, methanol, and methylamines [31,32]. Even though being biologically produced, methane can harm the environment if released into the atmosphere, due to its strong greenhouse warming potential [33], but when captured, it can serve as a promising renewable energy source.

### 2.2. Aerobic C1 Assimilation

In contrast to anaerobic C1-utilizing microorganisms, aerobic ones utilize a broader spectrum of carbon assimilation pathways. The most prominent example is the ATP-intensive Calvin–Benson–Bassham (CBB) cycle, which is widely distributed among photo- and chemoautotrophic organisms. The model organism *Cupriavidus necator* assimilates CO_2_ or formate and has been engineered for heterologous product formation and improved autotrophic growth [34]. Moreover, the CBB cycle has been successfully introduced into the heterotrophic organism *E. coli*, enabling autotrophic growth in a key biotechnological host [35]. This marks a major step toward sustainable conversion of CO_2_ into valuable chemicals.

Methylotrophic yeasts and bacteria (e.g., *Komagataella phaffii*, *Bacillus methanolicus*, *Methylorubrum extorquens*) as well as synthetic methylotrophs [36] use the ribulose monophosphate (RuMP) or serine pathways to grow on methanol as a carbon and energy source and are promising hosts for recombinant protein production and small-molecule biosynthesis. Both the RuMP [37] and serine pathways [38] have been engineered in *E. coli*, enabling the organism to grow on sustainable C1-substrates and to produce various heterologous products [38,39] with rates comparable to native methylotrophs.

Methanotrophic bacteria (e.g., *Methylococcus capsulatus*, *Methylomicrobium buryatense*) oxidize methane to methanol and further assimilate it via the ribulose monophosphate or serine pathway [40,41]. They are exploited for the production of microbial protein and other cell mass-derived products such as the intracellular biopolymer polyhydroxybutyrate (PHB) [42], as being commercialized by, e.g., Newlight Technologies [43] and Mango Materials [44]. Microbial protein (single-cell protein, SCP) is a potential replacement for fishmeal in feed and for meat in food, where *Methylococcus capsulatus* is a well-known methanotrophic strain and *C. necator* a classic HOB (hydrogen oxidizing bacterium) for SCP. Another target product of methanotrophs is the osmolyte ectoine [45]. Economically, CH_4_ seems more attractive than H_2_ + CO_2_, since there is broad availability of it, including biogenic methane, whereas a broad H_2_ infrastructure is still at a very early stage.

Two fundamentally different types of limitations occur in native C1 pathways: anaerobic systems are restricted by ATP deficiency and therefore divert a substantial fraction of carbon into acetate as a by-product, whereas aerobic systems benefit from high ATP generation but are limited by the high ATP demand for CO_2_ fixation via the CBB cycle. As a consequence, neither route alone enables high yields of target molecules from CO_2_ and H_2_, highlighting the need for new metabolic engineering and process engineering approaches, hybrid or coupled process designs that combine the energetic advantage of both metabolic pathways. Recent breakthroughs in metabolic engineering have redirected C1 assimilation pathways toward industrially relevant products such as ethanol, acetate, 2,3-butanediol, succinic acid, and bioplastic precursors [46]. In parallel, synthetic C1-fixation pathways—such as the reductive glycine pathway (rGlyP) [47,48], or the CETCH cycle (crotonyl-CoA/ethylmalonyl-CoA/hydroxybutyryl-CoA) [49] are being explored to increase carbon efficiency and ATP yield. The rGlyP is among the most promising pathways to improve yields from formate or methanol compared to native pathways and supports growth in diverse hosts including *E. coli* and *C. necator*. In *C. necator*, rGlyP provided higher biomass yields on formate than the native CBB cycle [50], while in *E. coli* adaptive evolution enabled efficient growth on formate [51] or methanol [52,53]. The RuMP cycle has likewise been engineered in *E. coli*, achieving rapid growth surpassing native methylotrophs [54]. However, product yields from these systems remain low, with a current maxima of 10% [55] (formate-to-lactate, rGlyP) and 16% [56] (methanol-to-lactate, RuMP), emphasizing the need for further optimization.

## 3. Process Engineering Approaches

The coupled process design mentioned above combines C1 assimilation and target product formation. In two-stage systems anaerobic and aerobic organisms are cultivated in separate reactors. Typically, an acetogen first converts C1 substrates into acetate with high carbon efficiency, followed by an aerobic microbe that transforms acetate into more complex products. This strategy effectively turns the energy limitations of acetogens into a benefit, since acetate formation is no longer a loss of yield but becomes the desired intermediate substrate. As a result, the only major carbon loss originates from CO_2_ generated in the aerobic conversion step. The main trade-offs are higher technical complexity, costs associated with operating two reactors and pH control (the first reactor requires base addition, while the second needs acid, resulting in a significant amount of salt by-product), and challenges in maintaining sufficient concentrations of intermediate substrates. More recently, the implementation of aerobic and anaerobic phases in a single reactor has been demonstrated, reducing these barriers. In this setup, one microbe is initially grown aerobically to consume oxygen and create an anaerobic environment, after which an acetogen carries out C1 fixation. Subsequently, reintroduction of oxygen allows the aerobic partner to convert the intermediate into the target product [57]. Both microbes remained viable despite repeated transitions between oxic and anoxic conditions, suggesting a higher oxygen tolerance in acetogens than previously assumed. This opens the possibility of integrating sequential culture into simplified, single-reactor processes that combine the advantages of aerobic and anaerobic metabolism while minimizing technical barriers [57]. Another concept that aligns with the coupled process design described above is a gas fermentation process coupled via the gas phase to achieve complete carbon conversion of the feedstock into products [24]. Conventional methanotrophic processes typically reach only about 50% carbon yield [24,58]. By fixing that CO_2_ in a second fermenter, through adding H_2_ and using anaerobic microorganisms to make a second product, all supplied carbon can be converted into a product without residual CO_2_ emissions like in a classic fermentation process.

Some acetogenic bacteria, as well as methanogens, can use electrons supplied at a cathode to fix CO_2_ in a process called microbial electrosynthesis (MES) [59,60,61]. This is an emerging technology that improves the conversion of C1 substrates into multi-carbon products by microbes. MES couples an electrochemically polarized cathode to electroautotrophic microbes (notably acetogens, such as *Sporomusa ovata*, and certain hydrogenotrophic methanogens), which either accept electrons directly or use the hydrogen generated by the electrode to drive the Wood–Ljungdahl pathway and related CO_2_ reduction routes. This enables the synthesis of acetate, methane, and other reduced organics [62,63,64]. However, practical application still faces challenges. Electron transfer mechanisms (direct or mediated extracellular electron transfer versus H_2_-mediated transfer) remain unclear, productivity is lower than that of chemical electrosynthesis routes and methanogenesis can compete with acetogenesis in mixed systems. Current work is addressing these issues through inoculum enrichment, cathode engineering and scale-up strategies [59]. In summary, MES offers a promising way to improve the utilization of C1 substrates by microbes by supplying reducing power electrically. This process turns intermittent renewable electricity and CO_2_ into storable chemicals. However, scaling up this process to create robust, high-rate systems requires further advances in materials, microbiology, and reactor integration.

Traditionally, carbon capture and storage (CCS) strategies use the mineralization of CO_2_ into CaCO_3_ through geochemical processes [65], as an alternative to sequestration underground. Microbiologically induced calcium carbonate precipitation (MICP) offers a biotechnological complement. Here, microbes enable mineralization either by adsorbing cations onto negatively charged cell surface groups or by driving metabolic changes that increase carbonate saturation and promote nucleation [66]. Both autotrophic processes [67] (e.g., oxygenic and anoxygenic photosynthesis, non-methylotrophic methanogenesis) [68] and heterotrophic pathways [69] (linked to sulfur and nitrogen cycles) have been implicated in biologically mediated carbonate formation [70]. Beyond their role in permanent CO_2_ capture, these biotechnological processes have already been applied to sustainable construction practices, including bio-cementation [67], crack repair, and surface treatment of building materials [71] using bacteria and fungi [71]. These strategies demonstrate how microbial processes can mitigate greenhouse gas emissions (SDG 13: Climate Action) while also contributing to the development of sustainable infrastructure (SDGs 9 and 11: Industry, Innovation and Infrastructure; Sustainable Cities and Communities).

In addition to microbial and process-based approaches, enzyme-based strategies open new perspectives for CO_2_ fixation. A proof-of-concept 17-enzyme cascade based on the synthetic CETCH cycle [49] has shown that complex in vitro systems can be designed for this purpose, competing with natural CO_2_-fixing pathways. Protein engineering further illustrates how synthetic biology can accelerate progress in this area: for instance, the design of a novel glycolyl-CoA carboxylase, optimized through rational design and high-throughput screening, achieved a catalytic efficiency comparable to natural CO_2_-fixing enzymes [72]. In addition, enzymatic approaches can be coupled with electrochemical methods to enhance CO_2_ capture. Enzyme-based electro-catalytic production of formic acid from CO_2_ [73] is of significant research interest. Biocatalysts with high oxygen tolerance that can accept electrons from a cathode are being developed to make this route feasible. Bioelectrochemical CO_2_ reduction, using either purified enzymes or whole-cell biocatalysts [74,75], is characterized by high selectivity and energy efficiency. Enzyme-based CO_2_ fixation strategies [76] are closely aligned with several of the SDGs. By providing alternatives to fossil-based chemical processes, they directly contribute to SDG 9 (Industry, Innovation and Infrastructure) through the development of novel biotechnological platforms, while also advancing SDG 12 (Responsible Consumption and Production) by promoting resource-efficient and circular approaches to carbon utilization. Most importantly, they address SDG 13 (Climate Action) by offering sustainable routes for CO_2_ sequestration and mitigation of greenhouse gas emissions. While microbial systems currently dominate industrial applications of C1 biotechnology, enzyme-based processes represent an emerging research field and a potential next step toward highly efficient, modular bioprocesses.

## 4. Industrial Deployment and Commercial Milestones

The commercial maturity of C1 biotechnology has advanced rapidly and is exemplified by several industrial-scale operations. Current applications can be grouped into three domains:

(a) Gas-to-fuels and chemicals

Traditionally, syngas synthesis has relied on Fischer–Tropsch catalysis to yield fuels and bulk chemicals. In recent years, however, both academic research and industrial initiatives (e.g., LanzaTech, IneosBio, Coskata) have demonstrated the feasibility of syngas bioconversion by acetogenic fermentation as an alternative [77]. Despite this progress, the efficiency of microbial syngas utilization remains suboptimal and requires further improvement before it can rival established catalytic processes. Various industrially useful acetogenic bacteria have already been modified using synthetic biology, e.g., *C. ljungdahlii* [78,79], *C. autoethanogenum* [80,81], *A. woodii* [82] or *M. thermoacetica* [83,84]. LanzaTech has operational plants converting CO-rich steel mill off-gases into ethanol using acetogenic fermentation [85]. Their platform is also expanding toward isopropanol, jet fuel, and commodity chemicals [86]. The company has taken gas fermentation to full commercial maturity. In total, LanzaTech operates six commercial-scale plants worldwide, reaching a total capacity of approximately 300,000 t/year of ethanol, suppressing over 500,000 t of CO_2_ emissions annually [87]. Carbon Recycling International (CRI) converts CO_2_ and H_2_ into renewable methanol, a versatile platform chemical, with over 200,000 t/year of sustainable methanol production capacity [88]. Evonik, through initiatives such as *Carbon2Chem* [89] and *Rheticus* [90], explores biohybrid processes where industrial CO_2_ streams and renewable H_2_ are converted into higher-value chemicals.

Beyond these, electro-biohybrid systems coupling CO_2_ electroreduction with microbial catalysis are emerging as next-generation C1 biorefineries, synthesizing complex molecules directly from captured CO_2_ using renewable electricity. This approach promises novel, fully electrified C1 biorefineries. Using CO_2_ from concentrated (industrial) point sources makes more sense thermodynamically than direct air capture, due to the comparatively low concentrations in air of approximately 400 ppm [91]. To achieve reliable performance, it is necessary to ensure that microbial strains are robust against real-world CO_2_ streams, which may contain organic and inorganic contaminants. Avoiding a complex feed gas purification process is only feasible when strains can tolerate complex contaminant loads and maintain stable functionality under variable emission conditions. Langsdorf et al. have demonstrated that robust microbial strains can convert industrial exhaust gases containing impurities into bioplastics directly [92].

(b) Microbial protein and food/feed applications

Back in 1974, when the interest in microbial protein started, the association “UNICELPE” was founded [93] as the European Association of SCP Producers. Efforts stagnated due to cheap soy imports and have picked up speed in recent years. Today, several companies are working on bacterial SCP for use in animal feed as well as human food ingredients. For example, Calysta and Unibio produce protein-rich biomass targeting aquaculture, livestock feed and pet food from methane using aerobic methanotrophs. SCP in pet food has recently been commercialized [94]. Feed ingredients for companion animals are currently regarded as the “sweet spot” and entry point of the industry, offering attractive economics. Other players such as String Bio [95], Aerbio (formerly Deep Branch), White Dog Labs, KnipBio and Kiverdi also use methane as feedstock [96]. SolarFoods aim to produce SCP for food with hydrogen-oxidizing bacteria, by using CO_2_ from the HVAC (heating, ventilation, and air conditioning) system, which is preconcentrated to >1000 ppm [97]. They plan to seek GRAS (Generally Recognized as Safe) status assessment in the United States, and for novel food authorization in other key markets such as the UK and the European Union. Arkeon, who attempted to develop individual amino acids with archaea from CO_2_ and H_2_, recently went out of business [98], just like NovoNutrients [99]. Further startups in the space are CIRCE Biotechnologie [100] and Econutri [101], both from Austria, and German MicroHarvest. Recently, LanzaTech announced plans to expand their biorefining platform to produce LanzaTech Nutritional Protein (LNP) as the primary product by using a new microorganism in their existing bioreactor technology. Commercial facilities are being designed and expected to produce more than 30,000 MT of LNP per year. 0.5 MT per day of LNP is roughly the equivalent of feeding 9000 people with their daily intake of protein. The first of this site is expected to be operational by 2028 [102].

(c) Bioplastics and materials

Companies such as Mango Materials (Yopp™) [103] and Newlight technologies (Aircarbon™) [104] commercially produce PHAs from methane using gas fermentation. They offer a biodegradable alternative to fossil-based plastics. PHA are fully biodegradable, also in the marine environment, and very versatile; hence, they are often considered the “sleeping giant” of the bioplastics industry. Such materials not only reduce plastic pollution, including the avoidance of persistent micro- and nano-plastics, but also align with global efforts toward circular bioeconomy models. Several C1-based processes have moved beyond proof-of-concept to large-scale operation, with a growing innovation pipeline. Gas fermentation significantly reduces land and water use compared to soy or fishmeal, and SCP has been successfully tested for various target animals [105,106,107]. Investment incentives from the European Innovation Council, Green Deal or InvestEU Program, the U.S. Inflation Reduction Act, and Clean Fuel Standards are critical to enable commercialization of C1 processes, alongside funding for pilot-scale projects and regulatory approval. The Pilot4U database [108] lists accessible gas fermentation facilities. Efficiency, downstream processing costs, consumer acceptance (especially for food SCP), and regulatory hurdles (e.g., Novel Food, Regulation (EC) No 258/97 in the EU) remain significant barriers.

## 5. Technical Challenges and R&D Directions

Compared to conventional sugar-based fermentation, gas fermentation is characterized by low solubility of the feed gases in the aqueous fermentation medium [109], necessitating specialized fermenter designs such as bubble column, air lift and loop reactors. Particularly loop reactors allow for a higher gas conversion rate compared to conventional stirred tank reactors [42]. In gas fermentation, since the biomass density tends to be almost an order of magnitude lower than in sugar-based fermentation, more efforts are needed in downstream processing, similar to microalgae production. The complex downstream process also explains part of the high manufacturing costs of microalgae, resulting in a minimum selling price that is prohibitive for interesting target applications. Furthermore, explosive atmospheres have to be dealt with in several cases, such as with methanotrophs [CH_4_ and O_2_] and hydrogen-oxidizing bacteria [H_2_ and O_2_], particularly in the headspace (degassing section). On the positive side, gases can be purified comparatively easily. Hence, contamination in raw materials poses less of an issue; heavy metals and unwanted organic compounds can be removed from the gases. These steps tend to be more complex in the route of lignocellulosic biomass hydrolysis, which is the “classic” 2G route for biofuels and biomaterials. Gas fermentation, through anaerobic digestion of (wet) and gasification of (dry) biomass can access a broader feedstock base than enzymatic hydrolysis, positioning it as a more scalable platform technology. In some cases of gas fermentation, monoseptic operation may not be required, particularly when extremophiles are used. Mixed culture operations tend to be cheaper, in both CAPEX and OPEX, compared to single-culture processes. Mixed consortia are typically more robust than pure cultures under fluctuations in process conditions of feedstock composition. Continuous processes allow for higher productivities than batch operations, and they are easier to automate. However, economies of scale are always a topic of concern, as large plants (>5000 tons/year) are needed for low-value end products such as protein and bioplastics, and even larger facilities are required for biofuels and chemical building blocks, which tend to have even lower market prices but comparable manufacturing costs. Specialty compounds can be produced economically in smaller installations. Side product valorization can improve process economics further, as well as carbon credits for the voluntary carbon market when feasible. An example of such a side product is protein-rich biomass after biopolymer extraction or after harvesting extracellular products such as ethanol. For carbon credits, the voluntary carbon market seems attractive, for gas fermentation projects that create avoided emissions or durable removals through long-lived products (CCU, CCS).

Although substantial progress has been made in gas fermentation, including strain selection, conditioning and engineering, fermenter design, and the development of the fermentation and downstream processes, as well as the development of products, several technical challenges remain:

- Gas–liquid mass transfer limitations hinder productivity in gas fermentation due to the low solubility of CH_4_, CO and H_2_ in aqueous media.
- Preventing microbial cross-contamination between anaerobic and aerobic units.
- In case of methylotrophs and formatotrophs, toxicity at higher concentrations of methanol and formate restricts product titers and strain robustness.
- Genetic toolkits for non-model organisms (e.g., acetogens and methanotrophs, as well as archaea) are still limited compared to biotechnological “work horses” like *E. coli* or *S. cerevisiae*.
- Process scalability and safety, especially with flammable gases like CH_4_ and H_2_, require tailored reactor designs and risk mitigation strategies.
- Industry fragmentation with comparatively few players leading to stand-alone, in-house technical solutions, limiting fast, best practice industry advancement.

Ongoing research focuses on strain selection and conditioning, synthetic pathway design, electro-biotechnological integration [110,111], and life-cycle optimization [112] including side stream valorization. One such example is the use of the remaining biomass after intracellular product extraction for feeding purposes. Using waste streams for the main nutrients, e.g., P and N [113] from sewage and manure, would be desirable to not only close the “carbon cycle” but also to achieve nutrient circularity. Well-defined, stable industrial effluent streams, e.g., from dairy processing are more likely to be integrated into future P and N media than municipal sewage treatment outputs.

Notably, coupling C1 biotechnology with renewable energy, carbon capture (preferably from industrial point sources), and industrial side streams are expected to unlock a new generation of climate-positive (carbon-neutral or carbon-negative), resource-efficient production systems. Moreover, policy incentives, industrial uptake, and societal acceptance will determine whether C1 biotechnology can move from promising demonstrations to large-scale, climate-positive applications.

## 6. Integration into the Circular Bioeconomy

C1 biotechnology aligns with circular economy principles by converting waste streams—such as industrial off-gases, biogas, landfill gas or coalmine ventilation air or CO_2_ from fermentation (breweries) or industry (cement kilns, furnaces)—into feedstocks for new materials, feed and food. It enables a non-sugar bioeconomy, mitigating the food–fuel–feed conflict and reducing dependency on agricultural inputs. CO_2_ represents a highly sustainable feedstock, as its conversion into fuels and chemicals offers a pathway to mitigate greenhouse gas emissions (SDG 13). While direct air capture remains costly and technically challenging—and always thermodynamically disadvantageous—the use of concentrated industrial CO_2_ streams offers a more feasible route (SDG 12). Power-2-X technologies [114] point towards a good potential for integration by coupling renewable electricity with CO_2_ utilization. For biogas producers, C1 biotechnology offers an alternative outlet for valorization, allowing for potentially more stable and higher prices than the volatile energy market, provided that they reach the required output size for a gas fermentation facility.

In summary, the state-of-the-art in C1 biotechnology has moved decisively beyond proof-of-concept, with several companies having reached maturity and a few dozen startups pushing forward. The field is now entering industrial maturity in selected applications, with a robust innovation pipeline promising deeper integration into sustainable value chains. By valorizing C1-rich waste streams, C1 biotechnology integrates seamlessly into circular value chains and supports climate-positive production systems.

## 7. Contribution of C1 Biotechnology to Achieving the SDG

While the technological feasibility of C1 platforms is well established, their broader relevance lies in their potential contribution to global sustainability goals. C1 biotechnology holds significant potential to contribute to the realization of the SDGs by addressing core challenges at the intersection of climate change, resource efficiency, food security, and industrial transformation, at a scale large enough to make a real difference. Any solution for (bio)fuels, (bio)plastics, feed, food or commodity chemicals must be massively scalable to replace existing technologies in a meaningful way. Unlike traditional biotechnological approaches that rely on carbohydrate-rich crops, C1 platforms utilize non-edible, one-carbon substrates that are either waste-derived or can be synthesized from renewable energy. This makes C1 biotechnology intrinsically aligned with circular economy principles and climate mitigation strategies. Renewable carbon can be converted into a wide range of products, as fermentation processes offer greater selectivity than catalytic and thermocatalytic processes—which is particularly attractive for complex target products such as proteins and polymers.

Compared to (enzymatic) hydrolysis of waste biomass, which is costly and yields complex reaction mixtures, anaerobic fermentation or gasification leads to a very pure gas stream for fermentation, virtually independent on the feedstock. These gaseous raw materials can either be generated on site using mature, low-cost technologies or transported via existing pipeline infrastructure. Several C1-based processes are already operational at pilot and commercial scales, demonstrating tangible contributions to SDGs such as Climate Action (SDG 13), Affordable and Clean Energy (SDG 7), Industry, Innovation and Infrastructure (SDG 9), and Responsible Consumption and Production (SDG 12). Gas fermentation has enabled the transformation of industrial emissions into bioethanol [115] and sustainable aviation fuels [116], providing low-carbon alternatives to petrochemicals. Methane- and methanol-based fermentation systems are being used to produce microbial protein, significantly reducing land and water use while offering novel solutions to Zero Hunger (SDG 2) and Life on Land (SDG 15). Beyond direct gas fermentation to ethanol or organic acids, C1 biotechnology offers a unique opportunity to route carbon through acetate as an intermediate for SCP production. Coupling anaerobic gas fermentation by acetogens with aerobic fermentation by oleaginous yeasts such as *Y. lipolytica* enables efficient carbon channeling into protein-rich biomass with tailored amino acid profiles. pH-regulated feeding strategies enable the simultaneous supply of acetate and control of the process, thereby minimizing inhibitory effects and improving yield. Such two-stage processes can valorize industrial off-gases into animal feed or food ingredients with an order-of-magnitude lower land and water footprint compared to soybean meal, thereby directly advancing SDGs 2, 3, 14 and 15. Despite this progress, significant implementation gaps remain. In addition to the regulatory and economic barriers mentioned above, coupled C1 processes also face distinct technical challenges (see Section 5). Addressing these constraints will require novel reactor configurations, optimized feeding strategies, and modular process designs that allow flexible scaling. Regulatory barriers (e.g., waste-to-food [117], Novel Food), technological bottlenecks (e.g., gas–liquid mass transfer, product titers, strain robustness [118]), and cost competitiveness limit broader deployment. Compared to classic fermentation, fewer standardized equipment solutions are available, and engineering partners have less practical experience to draw from. Gas fermentation companies typically develop their own technologies and keep them proprietary, slowing down widespread technology transfer.

Furthermore, integration into regional bioeconomy strategies and food/feed safety approval are critical to unlocking the full potential of C1-based solutions for Good Health and Well-Being (SDG 3) and Decent Work and Economic Growth (SDG 8). To assess the current state and strategic relevance of C1 biotechnology across the SDGs, we provide the following structured overview (Table 1 and Table 2).

This overview illustrates that while C1 biotechnology is already contributing to several SDGs, its full systemic potential remains underexploited. Key levers to unlock this potential will require policy incentives, effective carbon pricing mechanisms, integration into bioeconomy strategies, and streamlined regulatory pathways for food and feed applications. Israel and Singapore have evolved into vibrant hubs for alternative protein companies, areas where Europe and the United States have room to catch up. If the above-mentioned barriers are addressed in the coming years, C1 biotechnology could emerge as a transformative driver in achieving the SDGs, especially in areas where conventional bio-based or fossil-based pathways cannot deliver the necessary impact.

With Table 2, this article presents a comprehensive crosswalk between C1 biotechnology capabilities and all 17 UN SDGs. While not all goals are equally influenced by C1 technologies, indirect contributions—especially through enabling systemic resilience, circularity, and equity—are strategically important and should be acknowledged in sustainability roadmaps and policy dialogues. The following discussion outlines the full depth of each SDG.

SDG 1—No Poverty

A critical success factor of the circular bioeconomy is low production costs, enabling affordable goods and supporting economic growth. The avoidance of starch and sugar as fermentation feedstock for large-volume products contributes to lower food prices by avoiding raw material competition. Expenses for food can consume a significant portion of the available household income particularly in developing countries. Affordable staple food, through alternative, microbial protein for feed and food, can be an important aspect. Also, the externalized costs from fossil plastics, which are on the order of 15 €/kg [120], are one order of magnitude larger than the selling price of commodity polyolefins. It is the impoverished part of populations that has to bear a significant part of these costs. Persistent micro- and nanoplastics cause health issues that aggravate poverty, which can be avoided by biodegradable plastics. Moreover, decentralized production along the value chain can create jobs in low-income regions and further fight poverty. Instead of relying on centralized agricultural supply chains, small-scale fermenters processing C1 substrates could enable local protein or material production, thereby enhancing regional resilience and reducing vulnerability to global market fluctuations.

SDG 2—Zero Hunger

Access to nutritious food, particularly proteins, is one of the many vital resources that needs to be addressed, given their vital role in human health. However, the reliance on traditional animal-based diets as the primary source of protein has revealed unsustainable environmental consequences. Through microbial protein production from C1 feedstocks, C1 biotechnology can directly address SDG 2 by providing affordable, resource-efficient alternatives to conventional crops and animal feed, that are decoupled from arable land, water availability, and weather extremes. This makes it a highly attractive option for ensuring food security in regions prone to droughts or poor soil conditions. Hunger is in part due to high food prices, and protein is the costliest bulk food component. SCP, derived from microbial cultures such as bacteria, yeasts, filamentous fungi, or microalgae, holds a protein content of 70% to up to >80% and is rich in essential nutrients, including growth factors, vitamins, minerals, and amino acids often lacking in traditional diets. SCP can replace fishmeal in aquaculture [121,122], avoiding overfishing. Aquaculture can be more sustainable than wild-caught fish. SCP can also be used in other feed formulations, such as for chicken [123], which is advantageous due to their FCR (feed conversion ratio) comparable to fish. In pet food, SCP presents an ingredient with reduced footprint potential. After nucleic acid removal, SCP also lends itself as alternative protein in food use [124], to replace meat. Traditional meat has been recognized as unsustainable, particularly when considering the growing demand [125,126].

SDG 3—Good Health and Well-Being

Avoiding persistent micro- and nanoplastics by switching to biodegradable polymers is a contribution of C1 fermentation to this goal. Also, the production of clean, safe proteins [127], high-value nutritional supplements (e.g., vitamin B_12_, omega-3 fatty acids) and specialty compounds in safe, controlled fermentations with a reduced carbon footprint and avoided emissions supports SDG 3. Such products can help prevent deficiencies in vulnerable populations.

SDG 4—Quality Education

It is undisputed that education can improve the life of an individual and strengthen society at large. Hence, an indirect impact of stimulating education and skills development in biotechnology, fermentation synthetic biology and green technologies can be postulated, stimulating the creation of new interdisciplinary education programs.

SDG 5—Gender Equality

Like other industries, C1 biotechnology can make an indirect contribution to SDG 5, by fostering gender equality through inclusive participation in emerging biotech fields. Diversity is increasingly recognized as beneficial for organizational success, and biotech firms can leverage diversity and inclusion to their advantage.

SDG 6—Clean Water and Sanitation

The fermentation of C1 compounds can very directly contribute to clean water and sanitation through a minimal water footprint compared to conventional agriculture and industry, avoided eutrophication and absence of persistent micro- and nanoplastic pollution. Additionally, processes can be coupled with wastewater treatment or nutrient recovery from sewage and manure, thereby avoiding eutrophication while simultaneously closing nitrogen and phosphorus cycles.

SDG 7—Affordable and Clean Energy

Syngas and methane can be converted into energy, power and heat, so there is competition over energy. However, there exists an excess of low value biomass and biomass waste and side streams so that it can safely be assumed that both renewable energy demands and feedstock requirements for C1 biotechnology can be met. There is a direct contribution to this SDG by gas fermentation into biofuels and synthetic fuels from, e.g., CO_2_ and H_2_, yielding ethanol for admixing to gasoline and SAF (sustainable aviation fuel). Due to their high energy density, liquid biofuels will continue to play an important role in the energy transition. Some of them can directly be used in fuel cells and microbial fuel cells, which provide further avenues for integrating energy and bioproduction. C1 biotechnologies are increasingly discussed in the context of Power-to-X strategies, where renewable electricity is coupled with CO_2_ and H_2_ conversion to yield fuels and chemicals, thus directly linking biotechnology with the energy transition.

SDG 8—Decent Work and Economic Growth

This sector creates new high-value jobs in green industry, which stimulates biotech sector growth, and creates employment and revenue opportunities along the entire value chain, supporting economic development in cities and communities.

SDG 9—Industry, Innovation and Infrastructure

Industry is a vital pillar of the economy, and one can see several national and international initiatives for “re-industrialization” in developed nations which now rely heavily on services. Industrial production is at the center of value creation, and C1 fermentation strengthens industrial capacity through novel bioreactor designs and carbon utilization technologies, and novel business models that replace conventional plastics with bioplastics and animal protein with microbial alternatives in feed and food.

SDG 10—Reduced Inequalities

By commercializing the utilization of waste biomass streams, enabling decentralized access to production, C1 biotechnology empowers the Global South and supports fair access to resources and technology.

SDG 11—Sustainable Cities and Communities

An ideal city would be self-sufficient, which is not feasible with today’s technologies given its small size and high population density. Yet integrating C1 fermentation into urban circular systems by municipal CO_2_ capture, or conversion of biowaste into alternative protein can help to make cities more self-sufficient and circular. Self-sufficiency has been targeted for energy so far [128], and becoming independent with regards to materials, feed and food, through waste streams being repurposed by gas fermentation, is a desirable, yet reachable vision.

SDG 12—Responsible Consumption and Production

C1 biotechnology directly supports the emissions-to-products path, and the concept of low-waste, circular biomanufacturing. Production can be considered “responsible” when it is resource-efficient and when the products follow the DNSH (do no significant harm) [129] and SSBD (safe and sustainable by design) [130] principles. Consumption in itself can never be truly sustainable, as it inherently is about “consuming”, but its impact can be rendered less. Consumption as a whole is aggravated by rising populations and economic development. However, instead of consuming finite fossil resources and generating waste, C1-based processes allow the conversion of CO_2_, CO, or CH_4_ into valuable commodities and specialty products, thereby closing the carbon loop. Another important contribution lies in side-stream valorization, such as the recovery and reuse of nitrogen, phosphorus, and trace minerals from wastewater or agricultural residues, further enhancing circularity. This approach contributes to resource-efficient biomanufacturing and reduces dependencies on land use, fertilizers, and fossil feedstocks.

SDG 13—Climate Action

SDG 13 represents one of the strongest arguments for integrating C1 biotechnology into the circular bioeconomy. The circular bioeconomy is based on renewable carbon, and thereby strives to develop more climate-friendly processes. By capturing CO_2_ and CH_4_ from industrial point sources or even directly from the atmosphere and converting them into useful products, C1 processes can go beyond climate neutrality and enable negative-emission technologies. The ideal bioprocess uses renewable energy and renewable carbon. When coupled with renewable energy, for example through the use of green hydrogen, C1 biotechnology allows the production of carbon-based fuels and materials without adding to the global carbon burden. However, the products need to be considered over their lifecycle impacts. If, for instance, bioplastic materials decompose in an uncontrolled way under anaerobic conditions (e.g., in nature, under water), the climate-potent CH_4_ can be released, undermining climate benefits.

SDG 14—Life Below Water

Reduction of overfishing by replacing fishmeal with alternative microbial protein, C1 biotechnology can contribute to healthier marine ecosystems. By lowering the demand for agricultural land and reducing nutrient overflow, C1-based protein and material production can indirectly protect aquatic biodiversity and reduce pressures on marine ecosystems.

SDG 15—Life on Land

Compared to sugar and starch as fermentation feedstocks, there is a drastically reduced land use, helping to avoid LUC (land use change) and ILUC (indirect land use change), including forest protection/conservation through the usage of non-agricultural biomass.

SDG 16—Peace, Justice and Strong Institutions

Transparent biotech governance, sustainability certification and trust in an emerging industry can bring the aforementioned positive side effects such as jobs and creation of value, which ultimately contribute to strengthen institutions and peace.

SDG 17—Partnerships for the Goals

The global R&D consortia, public–private technology development and new, emerging value chains, where partnerships are needed due to the complex, inter- and transdisciplinary nature of C1 biotechnology and the use of its products, highlight the role of partnerships in scaling C1 biotechnology. Collaborations between the Global North and South are particularly crucial to ensure fair and equal access to technologies, enabling global participation in emerging C1-based value chains.

## 8. Conclusions

C1 biotechnology has emerged as a transformative platform with the potential to address some of the most pressing sustainability challenges of our time. By enabling the conversion of non-edible, one-carbon feedstocks into fuels, chemicals, and proteins, it offers a fundamentally different trajectory compared to sugar-based or fossil-dependent production systems. In doing so, it circumvents key limitations of first-generation biotechnologies, particularly the conflict with food resources, land use constraints, and high greenhouse gas emissions. This technology is no longer confined to academic laboratories: industrial-scale implementations are already operational, particularly in gas fermentation for microbial single-cell protein and ethanol. These systems have demonstrated significant environmental benefits, such as typically >80% reductions in life-cycle CO_2_ emissions and drastic reductions in land and water use compared to conventional agricultural or petrochemical processes. As a result, C1 biotechnology contributes meaningfully to at least eight of the 17 UN Sustainable Development Goals (SDGs) directly, and indirectly supports several more. Its strongest impact is currently seen in SDG 7 (Clean Energy), SDG 9 (Industrial Innovation), SDG 12 (Sustainable Production), and SDG 13 (Climate Action).

Nevertheless, important challenges remain. Mass transfer limitations, product toxicity (in case of methanol), strain engineering complexity (for non-feed and non-food products), regulatory barriers (e.g., Novel Food approval), and limited public awareness currently constrain broader deployment. Moreover, the benefits of C1 biotechnology are not yet equitably distributed, with developing regions facing access and investment barriers. Addressing these issues will require concerted efforts in three areas:

1. Technology and process innovation: Improving productivity, substrate flexibility, and downstream processing while reducing costs and energy demand.
2. Policy and market frameworks: Introducing carbon pricing, renewable chemical quotas, and regulatory recognition of C1-derived products in food, feed, and fuel markets.
3. Education and global collaboration: Building inclusive capacity, facilitating knowledge transfer, and integrating C1 platforms into regional and local bioeconomy strategies.

As the 2030 deadline for the SDGs approaches, incremental improvements in conventional systems are unlikely to suffice. Transformative technologies like C1 biotechnology offer a unique opportunity to redesign industrial value chains, capture and reuse emissions, and produce vital goods with minimal environmental footprint. If coupled with renewable energy, carbon capture infrastructure, and supportive governance, C1 biotechnology can become a cornerstone of the post-fossil economy and a vital contributor to achieving a sustainable, equitable, and resilient global future.

## Figures and Tables

**Table 1:** Contribution of C1 biotechnology to its most relevant UN SDGs.

SDG	What Has Been Achieved	Current Gap	Full Potential of C1 Biotechnology	Comment
SDG 2: Zero Hunger	Microbial SCP from CH_4_ (methanotrophs) or CH_3_OH (methylotrophs) and from H_2_/CO_2_ (HOB) for food	Although bacteria are part of human diets (e.g., cheese), bacterial protein is Novel Food, i.e., there is limited regulatory approval; low public awareness due to the novelty of the technology	Displace soy and fishmeal in feed and meat in food; improve global protein access with low land/water use	For food, the nucleic acid content typically needs to be reduced. Some SCP has been on the market before as Novel Food (e.g., Quorn™) or has received Novel Food Approval (e.g., Fermotein™), which are fungal (yeast-based) SCP. Bacterial SCP has been suggested as a solution in a global food catastrophe [119].
	SCP for feed			For feed applications, the dried microbial biomass can be used directly in a certain percentage, as approved, e.g., by the European Feed Catalogue (Commission Regulation (EU) 2017/1017, amending EU 68/2013).
SDG 3: Good Health and Well-Being	Protein production without antibiotics or pesticides	Safety assessments and public acceptance needed	Production of clean, pathogen-free alternative proteins and nutrients; less risk for multi-resistant bacteria compared to meat production	Protein is the most expensive bulk component of our food, after carbohydrates and lipids (fat, oils), which all constitute approx. 1/3 in a balanced diet. A low-fat, low-carb diet can become less costly with the large availability of SCP, meeting the demand of a growing population.
SDG 7: Affordable and Clean Energy	Gas fermentation produces low-carbon ethanol and fuels	Low energy efficiency (50% for methanotrophs); high capital costs for the upstream and downstream process	Large-scale renewable fuel production from captured CO_2_ and syngas	While the gaseous feedstocks are obviously energetic themselves, liquid fuels for transportation, or fuel cells, are interesting because of their high energy density and compatibility with existing infrastructure. For biogas producers, gas fermentation offers an alternative route for sales and value creation other than energy.
SDG 8: Decent Work and Economic Growth	Emergence of high-tech biomanufacturing jobs (direct employment), as well as significant demand for products and services along the value chain, creating indirect jobs.	Investment and skills gap, especially in the Global South	Create green jobs in clean-tech bioindustry, rural development	The bioeconomy generally enables local value creation for the feedstock. Developed economies tend to have a good integration of waste and side streams, with less potential than developing economies. Fermentation facilities offer educated jobs.
SDG 9: Industry, Innovation and Infrastructure	Commercial-scale gas fermentation (e.g., LanzaTech, Calysta)	Technology transfer and scale-up challenges, product portfolio	Drive industrial decarbonization and novel infrastructure for bioeconomy	After the failed projects of ICI (Pruteen™) and Norferm (BioProtein™) decades ago, new market incumbents are entering the stage, such as SolarFoods (Solein™), Unibio (Bioprotein™) and Calysta (FeedKind™). An entire, new value chain is to be built for each product. Patent portfolios of the major players give testimony of the innovation pipeline.
SDG 12: Responsible Consumption and Production	Valorization of waste gases and CO_2_	Limited industrial symbiosis and supply chain integration	Close carbon loops, valorize emissions as feedstock, and reduce waste; use the products for safe and sustainable by design (SSbD) end products, and consider the entire lifecycle [irrespective of the production process]	SCP can be ideally stored and dosed, avoiding food waste (100% utilization of dry bacterial biomass; side stream valorization—residual biomass—after extraction of target products, e.g., monosodium glutamate (MSG) [54]. Bioplastic articles can be made sustainable by design, considering various end-of-life options. Bio-based chemicals (biofuels) and building blocks such as (divalent) acids and alcohols can have a strongly reduced carbon footprint. Production should be integrated, utilizing all biomass.
SDG 13: Climate Action	Life-cycle CO_2_ reductions > 80% shown in case studies	Not yet broadly deployed; policy support lacking	Negative-emission production of fuels and chemicals	Use of CO_2_ as a feedstock for bioprocesses, and capturing/utilizing fugitive methane emissions.
SDG 14: Life Below Water	Reduced need for fishmeal via microbial protein; less marine plastic pollution	No direct marine deployment yet	Alleviate pressure on wild fisheries via alternative aquafeed	SCP as sustainable aquaculture feed, taking burden from wild catch. Avoidance of detrimental long-term impacts from plastics by preventing persistent micro- and nanoplastic formation.
SDG 15: Life on Land	Reduced land use intensity compared to crop-based systems	Indirect impact still underreported	Enable land sparing, reduce deforestation linked to soy or palm	Replacing sugar as feedstock for bioprocesses relieves pressure for agricultural production, incl. demand for land, fertilizer, water and other inputs.
SDG 17: Partnerships for the Goals	Several R&D projects including transnational and international collaboration	Fragmented collaboration	Global technology transfer and collaborative scale-up platforms	Standards can support technology dissemination.

**Table 2:** Contribution of C1 biotechnology to the remaining UN SDGs.

SDG	What Has Been Achieved	Current Gap	Full Potential of C1 Biotechnology	Comment
SDG 1: No Poverty	Emerging bio-based industries in low-income regions (e.g., methane-based protein in Asia, Africa)	Lack of inclusive finance; rural deployment limited	Job creation, decentralized production from local gas/waste streams to support poverty alleviation	Local value creation for smallholders by biorefineries that use synthesis gas and methane from waste biomass streams, and affordable high-quality products such as alternative protein for local use.
SDG 4: Quality Education	C1 biotechnology incorporated into academic curricula and national and international research training networks	Low public and vocational education awareness	Stimulate STEM (science, technology, engineering and mathematics) education and green skills relevant to future bioeconomy jobs	Degrees in STEM subjects offer good job prospects, and qualified graduates can put their knowledge into practice, which also supports SDG 1.
SDG 5: Gender Equality	Some inclusive participation in biotech entrepreneurship and research consortia	Gender imbalance in STEM leadership persists	Opportunity to build gender-balanced leadership in emerging biotech sectors	The growing biotech sector offers opportunities for all, and it can benefit from diversity like any other industry.
SDG 6: Clean Water and Sanitation	Low water footprint of C1-based processes compared to agriculture	Quantification of water benefits still scarce	Reduce water use and pollution in protein/chemical production, enable water-conscious biomanufacturing	Conventional production comes with externalized costs, which are often neither known nor considered. SCP can avoid eutrophication, and biodegradable plastics do not produce persistent micro- and nanoplastics.
SDG 10: Reduced Inequalities	Potential for tech democratization via modular C1 platforms	Global access and IP-sharing limitations	Enable decentralized production in resource-poor regions using local H_2_/CO_2_/CH_4_ feedstocks	Taking pressure from agricultural production by the introduction of alternative feedstocks for biomanufacturing can support lower prices for basic staple foods. Strong bioeconomies can create wealth for entrepreneurs and other stakeholders, too.
SDG 11: Sustainable Cities and Communities	Industrial symbiosis with urban emitters (e.g., using municipal CO_2_ or biogas)	Few pilot-scale urban integrations	Embed C1 bioreactors into urban infrastructure, turning cities into carbon sinks and bioproduct hubs	Several cities experiment with future concepts such as “smart”, “green” or “digital” cities. Gas fermentation of urban waste streams allows for closed looped production in cities. Rural communities can become self-sufficient in selected products, without having to rely solely on external inputs.
SDG 16: Peace, Justice and Strong Institutions	Transparent EU-funded consortia with open science principles	Regulatory fragmentation; no formal governance frameworks	Promote ethical biotech governance, sustainability certification, and participatory innovation	A lack of affordable nutrition and reliance on foreign countries for basic materials such as (bio)plastics can be a dangerous breeding ground for conflict.
SDG 17: Partnerships for the Goals (already included above)	Multinational consortia in EU, US, and Asia; collaborations between academia and industry	Limited involvement of Global South; tech transfer bottlenecks	Facilitate North–South and South–South partnerships for sustainable biotech deployment	Gas fermentation is, once again, at the verge of commercialization. There are good opportunities for partnerships along the various value chains.

## Data Availability

No new data were created or analyzed in this study. Data sharing is not applicable to this article.
